# mTORC2 modulates feedback regulation of p38 MAPK activity via DUSP10/MKP5 to confer differential responses to PP242 in glioblastoma

**DOI:** 10.18632/genesandcancer.41

**Published:** 2014-11

**Authors:** Angelica Benavides-Serrato, Lauren Anderson, Brent Holmes, Cheri Cloninger, Nicholas Artinian, Tariq Bashir, Joseph Gera

**Affiliations:** ^1^ Department of Medicine, David Geffen School of Medicine, University of California, Los Angeles, CA, USA; ^2^ Jonnson Comprehensive Cancer Center, University of California, Los Angeles, CA, USA; ^3^ Molecular Biology Institute, University of California, Los Angeles, CA, USA; ^4^ Division of Hematology-Oncology, Greater Los Angeles Veterans Affairs Healthcare System, Los Angeles, CA, USA

**Keywords:** p38 MAPK, DUSP, mTOR

## Abstract

Dual-specificity phosphatases (DUSPs) dephosphorylate MAP kinases (MAPKs) resulting in their inactivation. Activation of MAPK signaling leads to enhanced DUSP expression, thus establishing feedback regulation of the MAPK pathway. The DUSPs are subject to regulation at the post-translational level via phosphorylation resulting in alterations of protein stability. Here we report that mTORC2 function leads to stabilization of the p38 MAPK phosphatase, DUSP10, thereby inhibiting p38 activity. We demonstrate that mTORC2 binds DUSP10 and phosphorylates DUSP10 on serine residues 224 and 230. These phosphorylation events block DUSP10 turnover resulting in inactivation of p38 signaling. We further show that insulin-stimulated PI3K/mTORC2 signaling regulates DUSP10 stability and p38 activity. Importantly, knockdown of DUSP10 or ectopic overexpression of nonphosphorylatable or phosphomimetic DUSP10 mutants was sufficient to confer differential mTOR kinase inhibitor responses to GBM cells *in vitro* and in murine xenografts. Finally, DUSP10 was shown to be overexpressed in a significant number of GBM patients. These data demonstrate the ability of the mTORC2 pathway to exert regulatory effects on the DUSP10/p38 feedback loop to control the cellular effects of mTOR kinase inhibitors in GBM and support the use of DUSP10 expression as a surrogate biomarker to predict responsiveness.

## INTRODUCTION

The p38 mitogen-activated protein kinases (MAPKs) are responsive to several stress stimuli such as UV irradiation, heat shock and cytokine exposure. They are involved in the processes of cellular differentiation, autophagy and apoptosis [1]. Dual-specificity MAPK kinase 3 and SAPK/ERK kinase activate p38 by phosphorylating threonine 180 and tyrosine 182 [2]. Conversely, the regulation of p38 signaling is dependent on the activity of several p38 phosphatases such as DUSP1 and DUSP10 which dephosphorylated these residues [3]. It is hypothesized that these DUSPs constitute a negative feedback loop which functions to establish a threshold of MAP kinase activation through preemptive dephosphorylation of MAP kinases [3, 4]. This function has been proposed for DUSP3 as well as with non-DUSP phosphatases such as PP2A [4].

The regulation of DUSP activity is tightly controlled post-translationally, as the half-lives of many of the DUSPs is only approximately 60 min [5]. This tight regulation is partially mediated by the phosphorylation of DUSPs by MAP kinases that block the turnover of DUSPs by proteasomes [3]. This regulation of DUSP stability, coupled with their relatively short protein half-life and high inducibility suggests that DUSPs are likely to function as an immediate-early-off-switch regulating MAP kinase signaling [3]. Whether other signaling cascades can affect DUSP expression post-translationally is not completely understood.

The mTOR kinase is a central regulator of cell growth and size [6]. Regulation of mTOR signaling plays a major role in cancer and also in the cellular responses to nutrients, mitogens and chemotherapeutic agents. mTOR is present in two functionally and structurally individual multiprotein complexes termed TOR complex 1 (TORC1) and TORC2 [7]. The mammalian TOR complex 1 (mTORC1) contains mTOR, mLST8 and Raptor and is rapamycin sensitive. mTORC2 consists of Rictor, mSIN1, mLST8, PRR5 and mTOR and is rapamycin insensitive. Recently developed mTOR kinase inhibitors effectively block the activities of both complexes, thereby more dramatically inhibiting protein synthesis, suppressing AKT activation and inducing G_1_ arrest or apoptosis in tumor cells as compared to rapamycin [8]. mTORC1 signaling results in the phosphorylation of its downstream effectors p70/S6K and 4E-BP1 [9]. Activation of mTORC2 leads to phosphorylation of the AKT kinase at serine 473 resulting in AKT's full activation [10].

mTOR signaling plays a critical role in the cellular response to various stresses [11]. mTORC1 regulates growth and cap-dependent mRNA translation via effects on p70S6K and 4E-BP1 and inhibits autophagy [12]. Signaling inputs sensing the relative availability of amino acids have been demonstrated to enter the mTORC1 pathway from the lysososome via the Rag GTPases [13]. Muscle contraction or fluid and sheer stresses induce p70/S6K activity, while heat shock, reactive oxygen intermediates, osmotic stresses and DNA damage generally decrease p70/S6K function [14]. The mechanisms by which these latter stimuli signal to the mTORCs are not well understood.

In this report we demonstrate that Rictor and DUSP10 interact in yeast and mammalian cells and that DUSP10 is a substrate for mTORC2. We identified the phosphosites on DUSP10 and demonstrate that phosphorylation of these residues alters DUSP10 protein stability by blocking its degradation. We further demonstrate that stimulation of PI3K signaling, leading to mTORC2 activation, stabilizes DUSP10 and results in suppression of p38 MAP kinase activity supporting the ability of the mTOR pathway to regulate DUSP10 expression post-translationally. Finally, we show that enforced expression of either an mTORC2 nonphosphorylatable or phosphomimetic DUSP10 phosphosite mutant is sufficient to alter cellular responsiveness to mTOR kinase inhibitors.

## RESULTS

### DUSP10 interacts with the Rictor component of mTORC2

**Figure 1:**
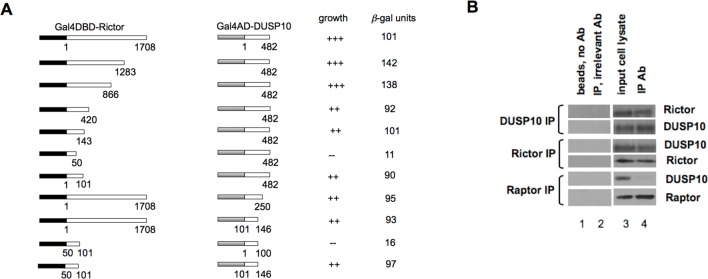
Interaction of Rictor and DUSP10 in yeast and mammalian cells A). The indicated deletion mutants of Gal4DBD-Rictor or Gal4AD-DUSP10 were cotransfected into AH109 cells to determine whether an interaction between the proteins was detectable via activation of the *HIS3* reporter (+++, strong growth; ++, moderate growth; -, no growth). Colonies which grew were assayed for *β*-gal activity. B) Rictor, Raptor or DUSP10 was immunoprecipitated from U87 cells and precipitates subjected to Western analysis for the indicated proteins. Lane 1, beads, no antibody; Lane 2, immunoprecipitation with an irrelevant antibody (control IgG); Lane 3, input cell lysate; Lane 4, indicated immunoprecipitate probed with antibodies for the indicated proteins. As a control, in Raptor immunoprecipitates DUSP10 was not detected.

In a previous large-scale yeast two-hybrid screen for interactors of the mTORC2 regulatory subunit Rictor, we identified Hsp70 as a Rictor interacting protein which was demonstrated to be required for supracomplex assembly and activity under hyperthermic conditions [17]. To extend our screening efforts, we prepared cDNA libraries from cell lines which overexpressed Rictor and were dependent on mTORC2 signaling for growth in an effort to capture additional Rictor interactors which may have not been represented well in the libraries utilized in our previous study. Utilizing these cDNA libraries we identified most of the known Rictor interacting proteins as well as novel binding proteins. A majority of the total recovered clones identified the major cytoplasmic phosphatase for p38 MAPK, DUSP10 (MKP5) as a Rictor binding partner. Several other genetic interactors were identified and are listed in Table 1. To determine which regions were involved in the binding of Rictor to DUSP10 we generated a set of deletion mutants shown in figure 1A. Rictor or DUSP10 were fused to either the Gal4-activation domain (AD) or Gal4-DNA binding domain (DBD) and these constructs were introduced into the yeast two hybrid strain AH109, plated on selective media to assess growth and the relative strength of the interactions were determined by liquid β-galactosidase assays. As shown, a region close to the N-terminus of Rictor (a.a. 50-101) was required for binding with the full-length DUSP10- AD fusion. A region of DUSP10, N-terminal to the MAP kinase binding domain (a.a. 101-146), was required for substantial reporter activity with full-length Rictor. Fusions of only the interacting domains within Rictor and DUSP10 to the Gal4-DBD or AD, respectively, were sufficient to mediate a robust interaction and led to significant growth and high levels of β-gal activity under selective conditions. We also confirmed the interaction of endogenous Rictor with DUSP10 in mammalian cells via co-immunoprecipitation experiments (figure 1B). As shown, anti-DUSP10 antibodies were able to effectively co-immunoprecipitate Rictor, as well as in the reciprocal fashion; Rictor antibodies co-immunoprecipitated DUSP10, while antibodies to the mTORC1 subunit Raptor did not, supporting the notion that DUSP10 is specifically associated with mTORC2. These data suggested that domains within the N-terminal half of Rictor and immediately N-terminal to the MAP kinase binding domain within DUSP10 mediate the interaction of these two proteins and that the full-length endogenous proteins interact in mammalian cells. Moreover, as purified Rictor did not affect DUSP10 phosphatase activity directly, as determined via an *in vitro* phosphatase assay (not shown) we hypothesized that DUSP10 may be a substrate for mTORC2 via interaction with Rictor.

**Table 1:** Genetic interactors identified in yeast two-hybrid screens utilizing Rictor as bait

Prey	Accession #	# of hits	Description
DUSP 10	NM_007207	32	Dual specificity phosphatase 10, MKP-5
CUL1	NM_003592.2	10	Cullin-1
PAK2	NM_002577.4	8	p21 protein (Cdc42/Rac)-activating kinase 2
AMOTL2	NM_091278683	7	Angiomotin-like 2
mTOR	NM_004958	7	Target of rapamycin kinase
YBX1	NM_004559	6	Y-box binding protein 1
mLST8	NM_022372	6	G protein β-subunit-like-protein, GβL
SIN1	NM_0241117	5	Mitogen-activated protein kinase associate protein 1, MAPKAP1
Hsp70	NM-005345	5	Heat shock 70 kDa protein 1A
BAT2	NM_080686	4	Proline-rich coiled-coil 2A, PRRC2A
ILK	NM_001278441	3	Integrin-linked kinase
SGK1	EU518415	3	Serum/glucocorticoid regulated kinase 1
SSH1L	NM_018984.3	3	Slingshot protein phosphatase 1
Protor-1	NM_001017528	2	Proline rich 5 ORF, PRR5

### DUSP10 is phosphorylated by mTORC2 at serines 224 and 230

**Figure 2:**
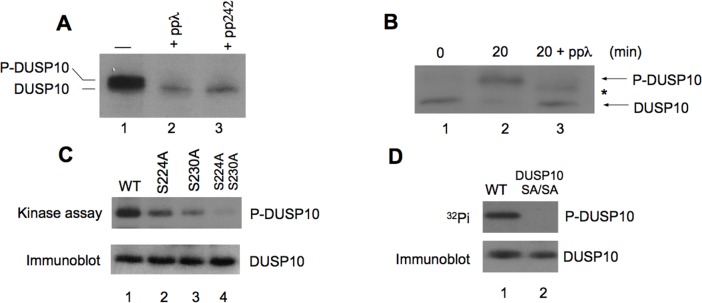
DUSP10 is phosphorylated by mTORC2 A). U87_Rictor_ cells harboring active mTORC2, display a slower migrating DUSP10 species (lane 1) which is eliminated by protein phosphatase lambda (ppλ) *in vitro* (lane 2) or by treating cells with PP242 (50 nM, 24 h) (lane 3). B). Immunoprecipitated mTORC2 phosphorylates recombinant DUSP10 *in vitro*. High-resolution SDS-PAGE of mTORC2 kinase reactions utilizing recombinant DUSP10 as a substrate and [γ32P]ATP for the indicated time (min) and treated with ppλ as shown. Phosphorylated and unphosphorylated species are as indicated, as well as partially dephosphorylated DUSP10 (asterisk). C). DUSP10 was mutated to produce the single mutants S224A and S230A and the double mutant S224A-S230A and treated with mTORC2 as described in (B). Wild-type (WT) DUSP10 and the mutants were subjected to an *in vitro* kinase assay with mTORC2 and [γ32P]ATP. Reactions were immunoprecipitated and detected by immunoblotting and autoradiography. D). U87_Rictor_ cells were transfected with expression plasmids encoding DUSP10 or the double mutant S224A-S230A (SA/SA) and 24 h following transfection cells were labeled with 32P (500 μCi/ml) in phosphate-free media for 4 h. DUSP10 was immunoprecipitated, resolved by SDS-PAGE and revealed by autoradiography (top) or immunoblotted (bottom). Results in A, B were performed three times with similar results.

An examination of the DUSP10 sequence identified two potential consensus mTOR phosphorylation sites [22] at serines 224 and 230 within the primary sequence. We also noted that in glioblastoma cell lines harboring differential mTORC2 activity as a result of ectopic Rictor overexpression [18], DUSP10 mobility was altered in SDS-PAGE analysis. In Rictor overexpressing U87 cells, containing active mTORC2, DUSP10 displayed reduced SDS-PAGE mobility. To determine whether the reduction in DUSP10 mobility was due to phosphorylation we treated cell extracts with lambda PP, which resulted in the elimination of the more slowly migrating species of DUSP10 (figure 2A). Treatment of these cells with PP242 similarly abolished expression of the slower migrating phosphorylated DUSP10 and occurred in a dose-dependent fashion (figure 2A, Supplementary figure S1). Immunoprecipitated mTORC2 phosphorylated recombinant DUSP10 *in vitro* and the phosphorylation was reversible after addition of lambda PP. These reactions were separated on high-resolution gels to clearly observe the alterations in DUSP10 mobility (figure 2B). Subsequently, we generated substitution mutants of DUSP10 at the candidate mTORC2 phosphorylation sites. Each serine residue was changed to alanine, either individually or in combination. *In vitro* kinase assays demonstrated that each single DUSP10 mutant exhibited reduced phosphorylation by immunoprecipitated mTORC2 and the double mutant DUSP10 (S224A, S230A), showed no phosphorylation (figure 2C). Moreover, in Rictor overexpressing U87 cells harboring activated mTORC2, the DUSP10 double mutant was not phosphorylated while wild-type DUSP10 displayed significant phosphorylation *in vivo* (figure 2D). These data demonstrate that mTORC2 is able to phosphorylate serines 224 and 230 on DUSP10.

### Differential mTORC2-dependent stability of DUSP10

**Figure 3:**
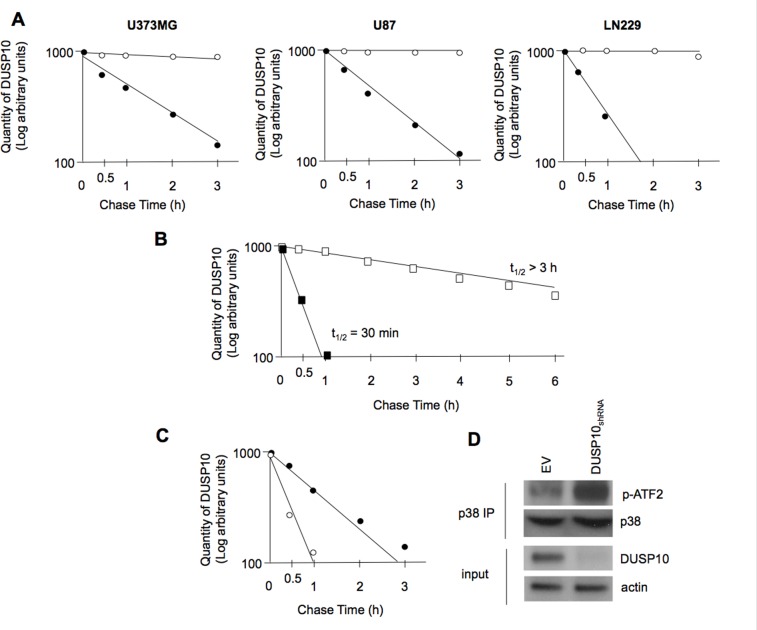
Half-life of DUSP10 is altered in response to modulation of mTORC2 A). Basal half-life of DUSP10 in U373MG (left panel), U87 (middle panel) and LN229 (left panel) glioblastoma cells. Cells were pulsed with ^35^S-methionine and DUSP10 levels monitored as described in the Methods section. Solid circles are in the absence, while open circles are in presence of the proteosome inhibitor MG-132 (25 nM) B). Differential stability of DUSP10 in U87_Rictor_ (open squares) cells versus U87_shRNARictor_ (solid squares) cells [18]. C). Destabilization of DUSP10 following PP242 exposure (10 nM, 6 h) in U373MG cells. Solid circles are in the absence of PP242, while open circles are in the presence of PP242. D). DUSP10 knockdown in U87 glioblastoma cells leads to enhanced p38 activity. Lysates from cells expressing shRNA targeting DUSP10 or empty vector (EV) were immunoprecipitated for p38 and immunoprecipitates subjected to an *in vitro* kinase assay using ATF2 as a substrate. Phosphorylated ATF2 was detected using phosphospecific antibodies. Input lysates were immunoblotted for the indicated proteins.

As a major mechanism of DUSP regulation involves regulated degradation via phosphorylation in a proteosome-dependent manner [23], we determined whether modulating mTORC2 activity would result in altered DUSP10 stability. As shown in figure 3A, in the glioblastoma lines U373MG, U87, and LN229 DUSP10 was degraded in a proteosome-dependent manner with a half-life of approximately 90 min, consistent with previous reports of the lability of other DUSPs [5, 24]. However, U87 cells in which ectopic overexpression of Rictor led to increased mTORC2 activity [18], DUSP10 was significantly stabilized (t_12_ > 3 h) while in cells expressing a shRNA targeting Rictor resulting in loss of mTORC2 activity, DUSP10 was very labile with a calculated half-life of only 30 min (figure 3B). As shown in figure 3C, DUSP10 was significantly destabilized following PP242 exposure with a calculated half-life of approximately 35 min. Moreover, we confirmed that in DUSP10 knockdown cells p38 MAPK activity is markedly increased, consistent with DUSP10 as being a major negative effector of p38 (figure 3D) [25]. These data suggest that enhanced mTORC2 activity is correlated with a marked increase in DUSP10 protein stability.

### Insulin-PI3K signaling stimulates mTORC2 mediated DUSP10 stabilization and p38 inactivation

**Figure 4:**
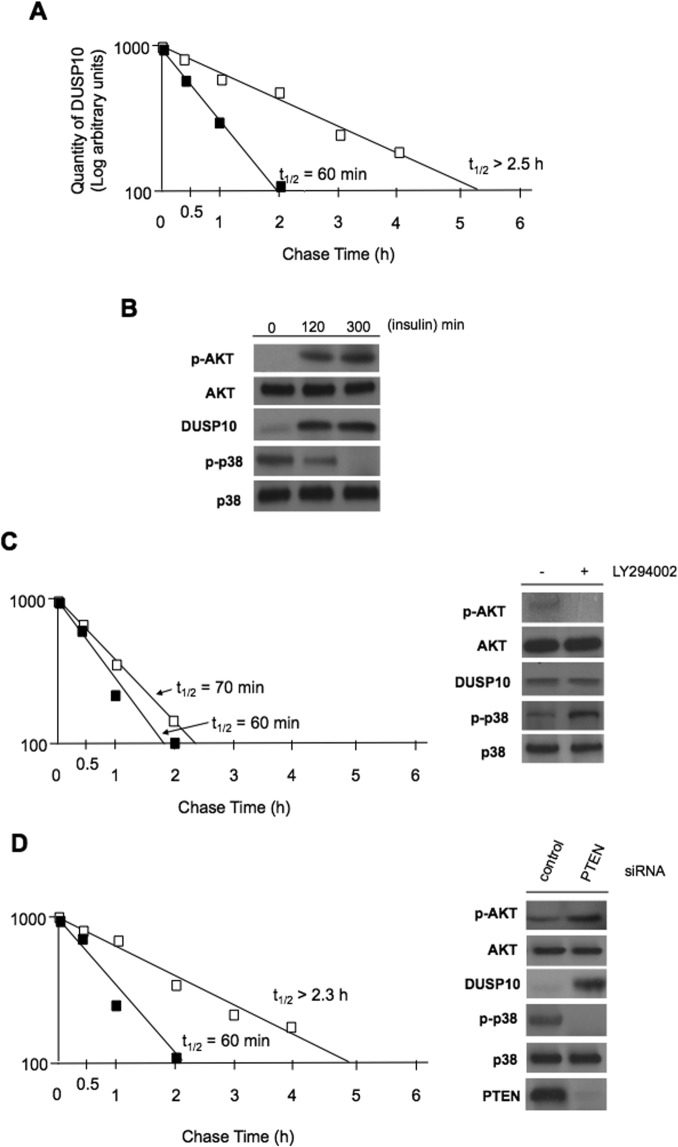
Insulin-PI3K signaling regulates DUSP10 stability and phosphorylated p38 levels A) Half-life of DUSP10 in U87 cells under basal (solid squares) and following insulin stimulation (open squares). B). Signaling effects of insulin stimulation in U87 cells. Insulin-stimulated cells (10 nM), treated for the indicated time points, were lysed and extracts immunoblotted for the indicated proteins. C). DUSP10 half-life (left panel) and signaling (right panel) in U87 cells treated with LY294002 (50 μM) (untreated, solid squares; LY294002 treated, open squares). D). siRNA-mediated knockdown of PTEN in LN229 cells leads to stabilization of DUSP10 (left panel; untreated solid squares; PTEN siRNA treated, open squares). Extracts from cells transfected with the indicated siRNA and immunoblotted for the indicated proteins (right panel).

Our data thusfar supported a signaling cascade in which activation of mTORC2 and subsequent phosphorylation of DUSP10 would lead to stabilization of the phosphatase and resulting p38 MAPK inactivation. We next investigated whether DUSP10 protein stability and p38 activity were regulated by insulin. Insulin is known to activate mTORC2 [26] and result in the activation of downstream effectors. Insulin treatment of U87 glioblastoma cells, which had been serum-starved for 18 h, resulted in the significant stabilization of DUSP10 as compared to control, unstimulated cells (figure 4A). Importantly, stabilization of DUSP10 correlated with increased phosphorylation serine 473 on endogenous AKT, a marker of mTORC2 activity (figure 4B). Furthermore, we observed a reduction in DUSP10 mobility and protein accumulation, as well as a marked reduction in phosphorylated p38 levels following insulin treatment consistent with the phosphorylation of DUSP10 by mTORC2 and resultant dephosphorylation of p38. These data demonstrate that insulin stabilizes DUSP10.

Insulin is known to activate mTORC2 via PI3K [27]. To ascertain whether PI3K signaling regulates DUSP10 stability, we examined whether DUSP10 degradation was affected upon blockade or stimulation of PI3K signaling. Inhibition of PI3K/mTORC2 signaling in cells treated with PI3K inhibitor LY294002 had no significant affect on DUSP10 stability and resulted in inhibition of phospho-Ser^473^-AKT and enhanced phospho-Thr^180^/Tyr^182^-p38 levels (figure 4C). DUSP10 SDS-PAGE mobility was also consistent with migration of the non-phosphorylated form of the phosphatase. In contrast, hyperactivation of PI3K signaling, accomplished by PTEN knockdown, a known negative regulator of PI3K signaling [28], markedly stabilized DUSP10 which accumulated to high levels in its phosphorylated form as compared to control cells (figure 4D). Again, the stabilization of DUSP10 upon PI3K hyperactivation was mirrored by a concomitant increase in the phosphorylation status of the endogenous mTORC2 substrate Ser^473^-AKT. Taken together these data suggest that insulin stimulates DUSP10 phosphorylation and stabilization in a physiological relevant manner dependent on PI3K/mTORC2 signaling.

### RNAi-mediated DUSP10 knockdown or overexpression of DUSP10 S224/S230 mutants alters responses of GBM cells to mTOR kinase inhibitors

**Figure 5:**
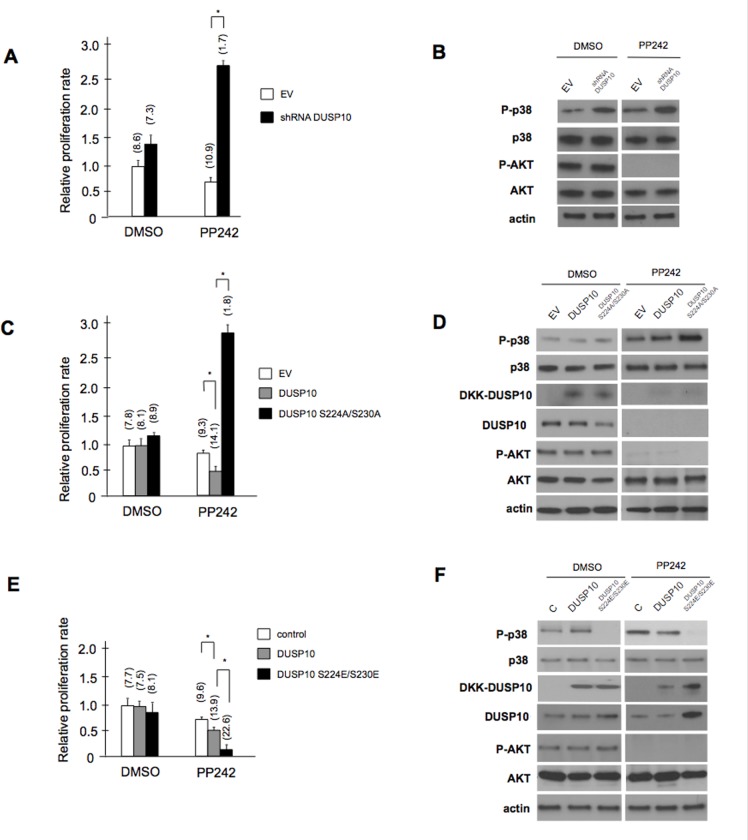
Effects of DUSP10 knockdown and nonphosphorylatable or phosphomimetic mutant overexpression on GBM responses to mTOR kinase inhibition A) Relative proliferation rates as determined from XTT proliferation assays of U87 cells expressing shRNA targeting DUSP10 as compared to empty vector (EV) transfected cells in the presence or absence of PP242 (25 nM, 24 h). Values in parentheses above bars correspond to percent apoptotic cells as determined via Annexin V staining. * *P* < 0.05 as determined by Student's *t*-test. B) Relative p38 and mTORC2 signaling in cells from (A). Extracts were prepared from the indicated lines and treatment groups and subjected to immunoblot analysis for the proteins shown. C&D) Effects of PP242 on proliferation, apoptosis and signaling in U87 cells overexpressing nonphosphorylatable DUSP S224A/S230A. * *P* < 0.05. E&F) As in (C&D), except performed using cells overexpressing the phosphomimetic DUSP10 S224E/S230E mutant. * *P* < 0.05.

Having previously demonstrated that a major mode of mTOR inhibitor resistance in GBMs was dependent on the induction p38 MAPK signaling [21] and to expand on the possible functional significance of mTORC2-mediated phosphorylation of DUSP10, we initially examined whether the U87 line expressing the shRNA targeting DUSP10 (figure 3D) displayed an altered response to PP242 as compared to empty vector control transfected cells. As shown in figure 5A, cells in which DUSP10 had been knocked-down were significantly more resistant to PP242 as compared to controls as determined by an XTT proliferation assay. In addition, the percentage of apoptotic cells, determined by Annexin V staining, markedly decreased from control values (percent apoptotic cells shown above bars). As expected, knockdown of DUSP10 or, as previously observed, following PP242 exposure, resulted in elevated phospho-p38 levels (figure 5B). Moreover, PP242 treatment effectively inhibited mTORC2 activity as phospho-AKT levels were dramatically reduced. These results supported our previous observations that p38 MAPK activity mediated a resistance pathway to mTOR inhibitors in GBMs [21] and were consistent with the notion that negating the DUSP10 blockade to p38 activity should result in a resistant cell phenotype in the face of PP242 exposure. Subsequently, we examined whether forced overexpression of nonphosphorylatable (224A/230A) or phosphomimetic (224E/230E) mutants as compared to wild-type DUSP10, or empty vector controls, would have effects on PP242 sensitivity. As shown in figure 5C, XTT proliferation assays demonstrated that U87 GBM cells stably overexpressing DUSP10 224A/230A were significantly more resistant to PP242 as compared to cells overexpressing wild-type DUSP10, however we also observed that native DUSP10 significantly decreased resistance to the drug upon overexpression. Additionally, the number of apoptotic cells was significantly reduced in PP242-treated cells overexpressing the nonphosphorylatable mutant, while cells overexpressing native DUSP10 displayed a modest increase in annexin V-positive cell numbers. The nonphosphorylatable DUSP10 mutant expressing cells also displayed elevated levels of phosphorylated p38 and downregulated DUSP 10 expression, relative to empty vector alone-transduced cells or cells overexpressing native DUSP10 (figure 5D). Overexpression of the DUSP10 mutants was confirmed by immunoblotting for the DKK-tagged allele and a marked reduction in phospho-AKT levels in PP242-treated cells was observed demonstrating ablation of mTORC2 activity. DUSP10 levels were not detectable in PP242-treated lines consistent with the inhibition of mTORC2 activity. In U87 cells stably overexpressing the DUSP10 phosphomimetic mutant (224SE/230SE) we observed a significant increase in sensitivity to PP242 as compared to controls (figure 5E). PP242-induced apoptosis also increased markedly in the phosphomimetic overexpressing cells. As shown in figure 5F, p38 signaling was blunted in cells overexpressing the phosphomimetic mutant, which correlated with relative increases in the levels of detectable total DUSP10. We also determined the relative half-lives of both the nonphosphorylatable and phosphomimetic DUSP10 proteins and as shown in supplementary figure S2, these were comparable to the half-lives observed for endogenous DUSP10 in lines containing quiescent or elevated mTORC2 activity, respectively (see figure 3C). Moreover, the calculated half-life observed for endogenous DUSP10 following PP242 treatment was comparable to that which was seen for the phosphomimetic mutant under similar conditions (see figure 3C). These data confirm a specific role for DUSP10 and support the functional significance of mTORC2-mediated phosphorylation events in determining the response to mTOR kinase inhibitors.

### *In vivo* effects of PP242 on mutant DUSP10 expressing lines

**Figure 6:**
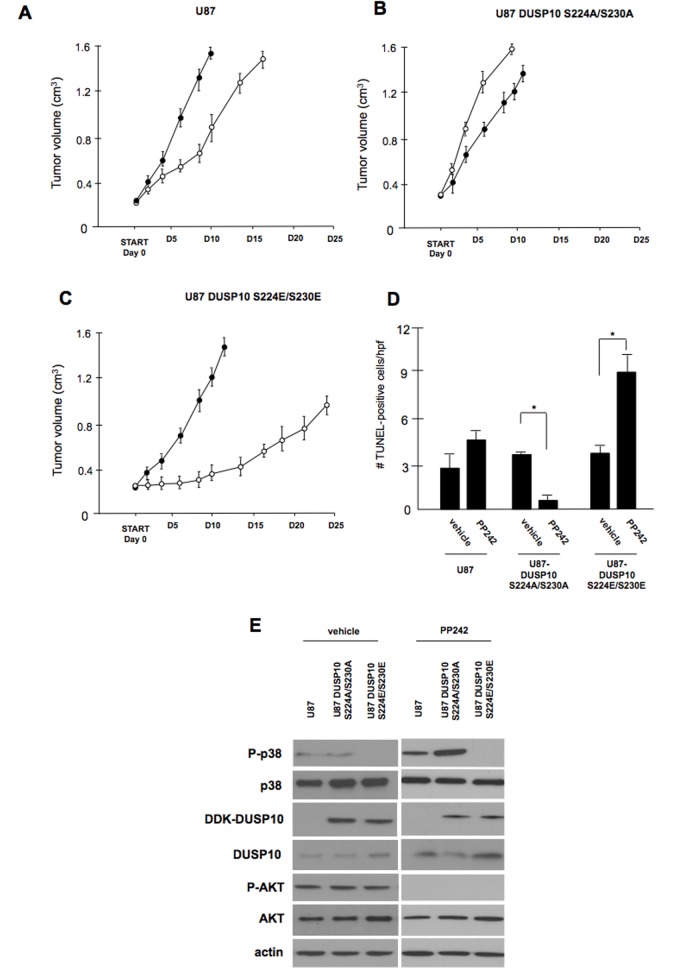
*In vivo* effects of PP242 on DUSP10 nonphosphorylatable or phosphomimetic expressing GBM lines A-C). Effects of PP242 treatment on U87, U87 DUSP10 S224A/S230A or U87 DUSP10 S224E/230E expressing lines in xenografts as indicated. Mice with established tumors (200 mm^3^) received either vehicle (closed circles) or PP242 (20 mg/kg/day, open circles) for 10 consecutive days and tumor growth was assessed every 2 days following initiation of treatment (start, day 0). D). Quantification of *in situ* TUNEL assay results from xenografts harvested at day 10 as indicated. The data are expressed as the number of positive apoptotic bodies divided by high power field (hpf; 10-12 hpf/tumor). Values are the means + SD. * *P* < 0.05. E). Effects of PP242 therapy on signaling pathways from day 10 harvested xenografts from the indicated lines. Immunoblot analysis was performed for the indicated proteins.

To ascertain what effects PP242 would have on the DUSP10 mutant overexpressing cell lines *in vivo* we performed xenografts experiments in SCID mice. Parental U87, U87-DUSP10 S224A/S320A (nonphosphorylatable) and U87-DUSP10 S224E/S230E (phosphomimetic) expressing lines were used to establish subcutaneous tumors in the rear flanks of mice and upon reaching ~200 mm^3^ in size were treated with either vehicle or PP242 for 10 consecutive days beginning at day 0 (figure 6A-C). In tumor cells overexpressing the nonphosphorylatable DUSP10 mutant xenografts, growth was modestly enhanced by PP242 treatment. Apoptosis induction in these tumors, as monitored by TUNEL staining following the 10-day treatment course, also showed a modest yet significant decrease relative to vehicle treated mice (figure 6D). P38 signaling in these tumors was significantly induced following PP242 treatments while DUSP10 expression was reduced (figure 6E). Conversely, in tumors derived from the phosphomimetic DUSP10 overexpressing cells, PP242 treatment markedly reduced tumor size, increased apoptotic induction, blunted p38 signaling and increased DUSP10 expression. These data support the *in vitro* effects observed for DUSP10 in response to PP242 and confirm the importance of the DUSP10 S244 and S230 phosphorylations on mTOR kinase inhibitor responses *in vivo*.

### DUSP10 is overexpressed in GBM patients

To seek clinical validation of the signaling relationships observed, we determined whether DUSP10 expression was altered in GBM. We analyzed an independent set of 32 quick-frozen glioblastoma samples and 5 normal samples. Each tumor sample was confirmed histologically, extracts prepared and the total relative abundance of DUSP10, phospho-Ser-^473^-AKT and phospho-Thr^180^/Tyr^182^-p38 expressed determined by Western analysis. Additionally, we determined the phosphorylation status of DUSP10 via high-resolution SDS-PAGE mobility as before. As shown in Table 2, seventeen of 32 (53%) tumor samples exhibited markedly elevated DUSP10 expression, which we defined as greater then a 10-fold increase relative to mean levels expressed in normal brain. We also observed a significant direct correlation in samples containing elevated DUSP10 levels with increased mTORC2 activity as well as phosphorylation of DUSP10 (*P* < 0.01). Moreover, elevated DUSP10 expression was significantly inversely correlated with p38 activity (*P* < 0.01). These data indicate that DUSP10 protein levels and phosphorylation is increased in GBM tumors and support the signaling pathways delineated in the cell line experiments.

**Table 2:** Relative DUSP10 expression and phosphorylation status in 32 primary glioblastoma samples

Samples	Relative DUSP10 expression§	Phosphorylated DUSP10	Relative mTORC2 activityˆ	Relative p38 activity¥
Normal				
1	1.3	−	1.4	1.2
2	1.1	−	−†	−‡
3	1.3	−	1.5	1.1
4	1.2	−	1.3	1.7
5	1.1	−	1.6	1.2
GBM				
1	54.9*	+	39.7	10.6
2	1.1	−	0.1	26.4
3	60.2*	+	34.9	2.6
4	82.4*	+	23.2	5.1
5	5.6	−	0.5	39.7
6	45.9*	+	63.6	4.1
7	9.2	−	1.7	40.6
8	50.6*	+	25.4	2.0
9	40.1*	+	55.3	−
10	35.2*	+	17.7	3.7
11	56.3*	+	26.0	0.9
12	3.3	−	5.3	19.2
13	29.6*	+	41.2	2.7
14	6.7	−	5.7	26.4
15	2.3	−	1.0	35.8
16	17.9*	+	19.4	−
17	5.1	−	0.7	17.1
18	2.1	−	−	12.9
19	29.4*	+	37.6	0.1
20	1.4	−	0.3	36.3
21	30.5*	+	17.9	10.2
22	0.8	−	4.3	22.8
23	43.1*	+	45.0	9.5
24	27.1*	+	19.6	2.2
25	0.7	−	0.6	17.9
26	71.6*	+	22.9	6.5
27	25.3*	+	16.6	11.5
28	5.1	−	3.8	11.7
29	47.1*	+	19.4	0.8
30	2.8	−	0.2	29.7
31	4.7	−	2.9	19.6
32	0.9	−	1.3	26.2

Note: Five normal brain and 32 quick-frozen GBM samples were assessed for DUSP10 expression and phosphorylation as described in figure 2 and quantified. Seventeen of 32 tumor samples (53%) had markedly higher expression levels of DUSP10 and displayed migration patterns consistent with phosphorylated DUSP10 (+) relative to normal brain. mTORC2 and p38 activity was determined by monitoring phospho-Ser^473^-AKT and phospho-Thr^180^/Tyr^182^-p38, respectively.

† Undetectable phospho-Ser^473^-AKT

‡ Undetectable phospho-Thr^180^/Tyr^182^-p38.

§ DUSP10 expression > 2-fold above mean of normal brain.

* Markedly increased DUSP10 expression > 10-fold increase above mean of normal brain.

ˆ mTORC2 activity > 2-fold above mean of normal brain.

¥ p38 activity > 2-fold above mean of normal brain.

## DISCUSSION

The activation of MAP kinases is accomplished by phosphorylation of both threonine and tyrosine residues within the signature sequence TXY of the kinase domain VIII [1]. The upstream activators have been identified and characterized and these are specific for each class of MAP kinase [29]. These kinases are inactivated by the dephosphorylation of these residues, which are carried out by members of the dual-specificity phosphatases [3, 23]. Certain DUSPs are inducible, while others are stabilized or destabilized by phosphorylation. Phosphorylation of DUSPs can lead to their stabilization by attenuating ubiquitination and subsequent degradation. For example, phosphorylation via ERK stabilizes DUSP1 and increases its half-life, whereas it elicits DUSP3 degradation [5, 30]. Our work supports the hypothesis that mTORC2 phosphorylates DUSP10 resulting in its stabilization. While we cannot formally exclude the possibility that additional kinases may similarly phosphorylate DUSP10 leading to stabilization, this stabilization presumably leads to accumulation of the protein which promotes its ability to dephosphorylate and inactivate p38. As our previous studies implicated sustained activation of the p38 signaling pathway in mTOR inhibitor resistance [21], we further tested whether alterations in DUSP10 activity would affect PP242 GBM tumor cell responses. Our data also support the contention that mTOR kinase inhibition, with compounds such as PP242, disrupt the ability of mTORC2 to stabilize DUSP10, leading to its rapid turnover with a resultant increase in p38 signaling activity.

Previous data supports the involvement of the mTOR signaling pathway in the regulation of the ERK phosphatase DUSP6 [31]. ERK/mTOR phosphorylation of DUSP6 on serine 159 and ERK phosphorylation on serine 197 induced a significant reduction in DUSP6 half-life promoting a positive feedback loop in response to MAPK signaling [32]. In contrast, our data show that phosphorylation of DUSP10 via mTORC2 on serines 224 and 230 enhances DUSP10 protein stability and negatively regulates p38 activity in response to PI3K activation. These variations in DUSP protein stability highlight the complex regulation of DUSPs post-translationally and may be a result of distinct signaling pathway inputs, particularly in the above case, as mTOR phosphorylation of DUSP6 was shown to be rapamycin sensitive and thus likely to be mediated by mTORC1.

While our data support the regulation of DUSP10 turnover by mTORC2 we cannot rule out the possibility that phosphorylation at serines 224 and 230 also may have effects on substrate targeting. Although we observed nearly complete dephosphorylation of p38 following mTORC2-mediated phosphorylation of DUSP10 following knockdown of PTEN (see figure 4D), which is consistent with the substrate preference of DUSP10 for p38 relative to other MAPKs, these residues are within the kinase binding domain of DUSP10 and may alter the affinity of the MKP-MAPK interaction. Indeed, Dickinson *et al*., demonstrated that serine 58 phosphorylation within the kinase interaction motif of DUSP9 impaired its ability to interact with ERK and p38 [33]. Interestingly, acetylation of lysine 57 of DUSP1, which is also within close proximity to critical arginine residues within its kinase interaction motif, stimulated binding of p38 [34]. It is certainly possible that the mTORC2-mediated phosphorylation events on DUSP10 or additional post-translational modifications are able to regulate substrate specificity.

It has been observed that individual DUSPs may have either increased or decreased expression in different tumor types [23]. For example, some DUSPs exhibit loss of heterozygosity in some cancers with additional functional studies supporting potential roles as tumor suppressors, including DUSP4, DUSP6 and DUSP7 [35-37]. However, some DUSPs are associated with increased expression in cancer progression. For example, DUSP1 expression is increased in pancreatic cancer and shown that its downregulation leads to reduced tumorigenicity [38]. Additionally, DUSP6 has been shown to be upregulated in non-small-cell lung carcinomas exhibiting hyperactive receptor tyrosine kinase activity and Ras/Raf signaling and potentially functioning as a negative-feedback regulator of mitogenic signaling [39]. Our data demonstrate that DUSP10 is overexpressed in a significant number of GBM samples and whether it plays a role in gliomagenesis and/or progression will be the subject of future experiments. Additional studies have also linked DUSP activity to chemotherapeutic resistance [30]. DUSP1 has been demonstrated to sensitize cancer cells to cisplatin-induced apoptosis [40] and overexpression of DUSP6 in estrogen receptor-positive breast cancer cells confers resistance to tamoxifen [41]. However, loss of DUSP6 expression in ovarian cancer cells has been demonstrated to increase chemoresistance [42]. Our RNAi-mediated DUSP10 knockdown and S224/S230 mutant overexpression data clearly demonstrate that reduced DUSP10 expression/activity results in heightened resistance to mTOR kinase targeted therapy in GBM lines, while an increase in DUSP10 expression/activity had the opposing effect (see figure 5).

In conclusion, we have identified DUSP10 as a novel substrate for mTORC2 via its interaction with Rictor. mTORC2-mediated phosphorylation of DUSP10 resulted in significant stabilization of the phosphatase and correlated with inhibition of p38 activity. Moreover, modulating DUSP10 activity resulted in altered GBM tumor responses to PP242 and elevated DUSP10 expression was demonstrated in a significant number of GBM patient tumors. Our data would also predict sensitivity to mTOR kinase inhibitors of GBM tumors harboring elevated DUSP10 expression. Given the importance of mTOR and DUSP/MKP signaling in tumor growth and drug resistance, a further detailed understanding of the signaling relationships linking these two pathways is warranted to effectively target these signaling cascades.

## MATERIALS AND METHODS

### Plasmids, cell lines, GBM samples and reagents

U87 and LN229 cell lines were obtained from ATCC (Manassas, VA). U373MG cells were obtained from Sigma. DNA constructs composed of portions of Rictor and DUSP10 were generated by PCR and individually subcloned into pGB12 and pACT2, respectively. Antibodies to Rictor, Raptor, mTOR, phospho-Akt (Ser473), Akt, DUSP10, p38, phospho-p38 (Thr180/Tyr182) and phospho-ATF-2 (Thr71) were from Cell Signaling (Beverly, MA). Actin and rabbit IgG (isotype control) antibodies were from Santa Cruz Biotechnology. PTEN antibodies and protein phosphatase lambda were from Millipore. DKK antibodies were from Origene. All other reagents were obtained from Sigma. To generate the DUSP10 S224/S230 substitution mutants the full length DUSP10 cDNA cloned into pCMV6-Entry (OriGene Technologies) was mutagenized using the QuikChange® II Site-Directed Mutagenesis kit (Agilent Technologies) utilizing appropriate mutagenic primers according to the manufacturer. All plasmids were sequenced to verify the constructs. Recombinant wild-type and mutant DUSP10 was expressed and purified from HEK293 cells using anti-FLAG immunoaffinity column chromatography as previously described [15]. DUSP10 shRNA constructs were also obtained from OriGene cloned into the retroviral vector pRS. Cells stably expressing the knockdown constructs were selected via culture in puromycin following retroviral transduction according to the manufacture's protocol. siRNA targeting PTEN was obtained from Cell Signaling (SignalSilence^®^ PTEN siRNA I). Transfections were performed using Effectene^®^ transfection reagent according to the manufacturer (Qiagen). Flash-frozen normal brain and glioblastoma samples were obtained from the Cooperative Human Tissue Network (CHTN), National Cancer Institute (Western Division, Vanderbilt University Medical Center) under an approved Institutional Review Board protocol.

### Yeast two-hybrid analysis

The yeast two-hybrid assays to isolate and map Rictor interacting proteins and domains were performed using standard procedures [16]. The full-length human Rictor cDNA was cloned into pGB12 in frame with the Gal4 DNA-binding domain. This construct was used to transform AH109 cells to obtain a strain which expressed the GAL4DBD-Rictor fusion. This strain was used to screen human brain cDNA libraries prepared from mRNA isolated from U87 and T98G cells (lines overexpressing Rictor), which was reverse-transcribed, size-selected and cloned into pACT2 (BD Biosciences, Clontech). Liquid β-gal assays were performed as previously described [16].

### Immunoprecipitations and protein analysis

Immunoprecipitations and Western analyses were performed as previously described [17], except that TrueBlot™ reagents (Rockland) were used for DUSP10 immunoprecipitations to avoid IgG heavy chain masking. For DUSP10 half-life determinations, cells were cultured with ^35^S-methionine for 45 min followed by the addition of unlabeled methionine (1 μM). DUSP10 was immunoprecipitated at the indicated time points and imaged via autoradiography. DUSP10 bands were excised and counted in a scintillation counter for quantification. Data shown are the means from three independent experiments.

### *In vitro* kinase and phosphatase assays

mTORC2 *in vitro* kinase assays were performed as previously described [18] except performed in the presence of 50 μM ATP and [γ^32^P]ATP [2 μCi per sample] together with 2 ng of *in vitro* transcribed and translated wild-type or mutant DUSP10 as indicated. Reactions were terminated by addition of 1% SDS and 4 M urea and resolved by SDS-PAGE. p38 *in vitro* kinase assays were performed as described [19], utilizing recombinant ATF2 as a substrate and phosphorylation determined via immunoblotting with phospho-specific antibodies. DUSP10 phosphatase activity assays were performed using O-methyl fluorescein phosphate (OMFP) as previously described [20].

### Cell proliferation/apoptosis assays

Cells were seeded in 96-well plates and were treated with mTOR kinase inhibitor for 24 h as indicated in media containing 1% FBS. Relative proliferation to control and empty vector transfected cells with vehicle treatment was determined via an XTT Cell Proliferation Assay Kit II (Roche Diagnostics). Cells were incubated for 2 h following the addition of the tetrazolium salt XTT at 5% CO2 and 37˙ C and absorbances of control DMSO or PP242 treated cells was measured with a microplate reader (Bio-Rad) at 420-480 nm. Cells were stained for Annexin V using a fluorescein isothiocyanate-conjugated anti-Annexin V antibody (Abcam) and analyzed via flow cytometric measurements. For terminal deoxynucleotidyl transferase-mediated dUTP nick end labeling (TUNEL) staining of sections, slides were stained using the TACS-XL^®^ DAB *In situ* Apoptosis Detection kit (Trevigen) according to the manufacturer's instructions and counterstained with hematoxylin as previously described [21].

### *In vivo* studies

For murine xenografts experiments 1 × 10^6^ cells of the indicated lines were injected subcutaneously into the right flank of 4-wk-old SCID mice in 100 μl of Matrigel™, PBS 1:2 solution. After tumors reached ~200 mm^3^, mice were treated daily with 20 mg/kg PP242 for 10 days and tumors measured with an electronic caliper and volumes calculated using the formula *L x W^2^ x 0.5*, where *L* is the longest length, and *W* is the shortest length. Mice were euthanized after when tumor volumes of different lines reached statistical significance. Tumors were also harvested for immunoblot analyses of relevant signaling proteins. Mice were euthanized in accordance with approved institutional guidelines for animal welfare.

## SUPPLEMENTARY MATERIAL FIGURES



## References

[1] Cargnello M, Roux PP (2011). Activation and function of the MAPKs and their substrates, the MAPK-activated protein kinases. Microbiol Mol Biol Rev.

[2] Raingeaud J, Gupta S, Rogers JS, Dickens M, Han J, Ulevitch RJ, Davis RJ (1995). Pro-inflammatory cytokines and environmental stress cause p38 mitogen-activated protein kinase activation by dual phosphorylation on tyrosine and threonine. J Biol Chem.

[3] Huang CY, Tan TH (2012). DUSPs, to MAP kinases and beyond. Cell Biosci.

[4] Todd JL, Tanner KG, Denu JM (1999). Extracellular regulated kinases (ERK) 1 and ERK2 are authentic substrates for the dual-specificity protein-tyrosine phosphatase VHR. A novel role in down-regulating the ERK pathway. J Biol Chem.

[5] Brondello JM, Pouyssegur J, McKenzie FR (1999). Reduced MAP kinase phosphatase-1 degradation after p42/p44MAPK-dependent phosphorylation. Science.

[6] Laplante M, Sabatini DM (2012). mTOR signaling in growth control and disease. Cell.

[7] Wullschleger S, Loewith R, Hall MN (2006). TOR signaling in growth and metabolism. Cell.

[8] Feldman ME, Apsel B, Uotila A, Loewith R, Knight ZA, Ruggero D, Shokat KM (2009). Active-site inhibitors of mTOR target rapamycin-resistant outputs of mTORC1 and mTORC2. PLoS Biol.

[9] Ma XM, Blenis J (2009). Molecular mechanisms of mTOR-mediated translational control. Nat Rev Mol Cell Biol.

[10] Sarbassov DD, Guertin DA, Ali SM, Sabatini DM (2005). Phosphorylation and regulation of Akt/PKB by the rictor-mTOR complex. Science.

[11] Hung CM, Garcia-Haro L, Sparks CA, Guertin DA (2012). mTOR-dependent cell survival mechanisms. Cold Spring Harb Perspect Biol.

[12] Kroemer G, Marino G, Levine B (2010). Autophagy and the integrated stress response. Mol Cell.

[13] Jewell JL, Russell RC, Guan KL (2013). Amino acid signalling upstream of mTOR. Nat Rev Mol Cell Biol.

[14] Sengupta S, Peterson TR, Sabatini DM (2010). Regulation of the mTOR complex 1 pathway by nutrients, growth factors, and stress. Mol Cell.

[15] Tomomori-Sato C, Sato S, Conaway RC, Conaway JW (2013). Immunoaffinity purification of protein complexes from Mammalian cells. Methods Mol Biol.

[16] Gera JF, Hazbun TR, Fields S (2002). Array-based methods for identifying protein-protein and protein-nucleic acid interactions. Methods Enzymol.

[17] Martin J, Masri J, Bernath A, Nishimura RN, Gera J (2008). Hsp70 associates with Rictor and is required for mTORC2 formation and activity. Biochem Biophys Res Commun.

[18] Masri J, Bernath A, Martin J, Jo OD, Vartanian R, Funk A, Gera J (2007). mTORC2 activity is elevated in gliomas and promotes growth and cell motility via overexpression of rictor. Cancer Res.

[19] Shi Y, Sharma A, Wu H, Lichtenstein A, Gera J (2005). Cyclin D1 and c-myc internal ribosome entry site (IRES)-dependent translation is regulated by AKT activity and enhanced by rapamycin through a p38 MAPK- and ERK-dependent pathway. J Biol Chem.

[20] Rice RL, Rusnak JM, Yokokawa F, Yokokawa S, Messner DJ, Boynton AL, Wipf P, Lazo JS (1997). A targeted library of small-molecule, tyrosine, and dual-specificity phosphatase inhibitors derived from a rational core design and random side chain variation. Biochemistry.

[21] Cloninger C, Bernath A, Bashir T, Holmes B, Artinian N, Ruegg T, Anderson L, Masri J, Lichtenstein A, Gera J (2011). Inhibition of SAPK2/p38 enhances sensitivity to mTORC1 inhibition by blocking IRES-mediated translation initiation in glioblastoma. Molecular cancer therapeutics.

[22] Hsu PP, Kang SA, Rameseder J, Zhang Y, Ottina KA, Lim D, Peterson TR, Choi Y, Gray NS, Yaffe MB, Marto JA, Sabatini DM (2011). The mTOR-regulated phosphoproteome reveals a mechanism of mTORC1-mediated inhibition of growth factor signaling. Science.

[23] Patterson KI, Brummer T, O'Brien PM, Daly RJ (2009). Dual-specificity phosphatases: critical regulators with diverse cellular targets. Biochem J.

[24] Katagiri C, Masuda K, Urano T, Yamashita K, Araki Y, Kikuchi K, Shima H (2005). Phosphorylation of Ser-446 determines stability of MKP-7. J Biol Chem.

[25] Theodosiou A, Smith A, Gillieron C, Arkinstall S, Ashworth A (1999). MKP5, a new member of the MAP kinase phosphatase family, which selectively dephosphorylates stress-activated kinases. Oncogene.

[26] Yang Q, Inoki K, Ikenoue T, Guan KL (2006). Identification of Sin1 as an essential TORC2 component required for complex formation and kinase activity. Genes Dev.

[27] Zinzalla V, Stracka D, Oppliger W, Hall MN (2011). Activation of mTORC2 by association with the ribosome. Cell.

[28] Chalhoub N, Baker SJ (2009). PTEN and the PI3-kinase pathway in cancer. Annu Rev Pathol.

[29] Roux PP, Blenis J (2004). ERK and p38 MAPK-activated protein kinases: a family of protein kinases with diverse biological functions. Microbiol Mol Biol Rev.

[30] Caunt CJ, Keyse SM (2012). Dual-specificity MAP kinase phosphatases (MPKs): shaping the outcome of MAP kinase signaling. FEBS J.

[31] Bermudez O, Marchetti S, Pages G, Gimond C (2008). Post-translational regulation of the ERK phosphatase DUSP6/MKP3 by the mTOR pathway. Oncogene.

[32] Marchetti S, Gimond C, Chambard JC, Touboul T, Roux D, Pouyssegur J, Pages G (2005). Extracellular signal-regulated kinases phosphorylate mitogen-activated protein kinase phosphatase 3/DUSP6 at serines 159 and 197, two sites critical for its proteasomal degradation. Mol Cell Biol.

[33] Dickinson RJ, Delavaine L, Cejudo-Marin R, Stewart G, Staples CJ, Didmon MP, Trinidad AG, Alonso A, Pulido R, Keyse SM (2011). Phosphorylation of the kinase interaction motif in mitogen-activated protein (MAP) kinase phosphatase-4 mediates cross-talk between protein kinase A and MAP kinase signaling pathways. J Biol Chem.

[34] Cao W, Bao C, Padalko E, Lowenstein CJ (2008). Acetylation of mitogen-activated protein kinase phosphatase-1 inhibits Toll-like receptor signaling. J Exp Med.

[35] Furukawa T, Sunamura M, Motoi F, Matsuno S, Horii A (2003). Potential Tumor Suppressive Pathway Involving DUSP6/MKP-3 in Pancreatic Cancer. The American Journal of Pathology.

[36] Schullerus D, Herbers J, Chudek J, Kanamaru H, Kovacs G (1997). Loss of heterozygosity at chromosomes 8p, 9p, and 14q is associated with stage and grade of non-papillary renal cell carcinomas. The Journal of Pathology.

[37] Emmert-Buck MR, Vocke CD, Pozzatti RO, Duray PH, Jennings SB, Florence CD, Zhuang Z, Bostwick DG, Liotta LA, Linehan WM (1995). Allelic Loss on Chromosome 8p12-21 in Microdissected Prostatic Intraepithelial Neoplasia. Cancer Research.

[38] Liao Q, Guo J, Kleeff J, Zimmermann A, Büchler MW, Korc M, Friess H (2003). Down-regulation of the dual-specificity phosphatase MKP-1 suppresses tumorigenicity of pancreatic cancer cells. Gastroenterology.

[39] Sato M, Vaughan MB, Girard L, Peyton M, Lee W, Shames DS, Ramirez RD, Sunaga N, Gazdar AF, Shay JW, Minna JD (2006). Multiple Oncogenic Changes Are Not Sufficient to Confer a Full Malignant Phenotype on Human Bronchial Epithelial Cells. Cancer Research.

[40] Wang Z, Xu J, Zhou J-Y, Liu Y, Wu GS (2006). Mitogen-Activated Protein Kinase Phosphatase-1 Is Required for Cisplatin Resistance. Cancer Research.

[41] Cui Y, Parra I, Zhang M, Hilsenbeck SG, Tsimelzon A, Furukawa T, Horii A, Zhang Z-Y, Nicholson RI, Fuqua SAW (2006). Elevated Expression of Mitogen-Activated Protein Kinase Phosphatase 3 in Breast Tumors: A Mechanism of Tamoxifen Resistance. Cancer Research.

[42] Chan DW, Liu VWS, Tsao GSW, Yao K-M, Furukawa T, Chan KKL, Ngan HYS (2008). Loss of MKP3 mediated by oxidative stress enhances tumorigenicity and chemoresistance of ovarian cancer cells. Carcinogenesis.

