# Supplementary material for: mTORC2 modulates feedback regulation of p38 MAPK activity via DUSP10/MKP5 to confer differential responses to PP242 in glioblastoma
Source: Genes Cancer. 2014 Nov;5(11-12):393–406. doi: 10.18632/genesandcancer.41 (PMC4279437; doi:10.18632/genesandcancer.41)
Supplement: Supplementary file 1 [file ganc-05-393-s001.pdf]

mTORC2 modulates feedback regulation of p38 MAPK activity via DUSP10/MKP5 to confer differential responses to PP242 in glioblastoma

Supplementary Material

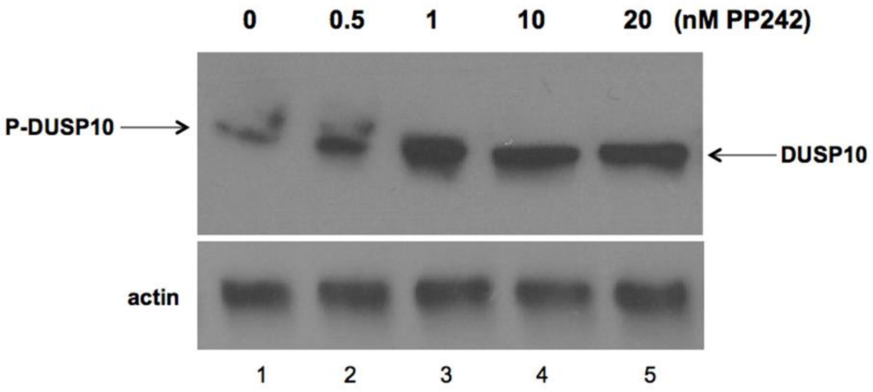

Supplementary Figure S1. Dose-dependent alteration of DUSP10 mobility in the presence of escalating concentrations of PP242. $U87_{Rictor}$ cells, harboring active mTORC2, were treated with the indicated doses of PP242 for 15 min and cell lysates immunoblotted for DUSP10 and actin. Differentially phosphorylated DUSP10 species are indicated by arrows.

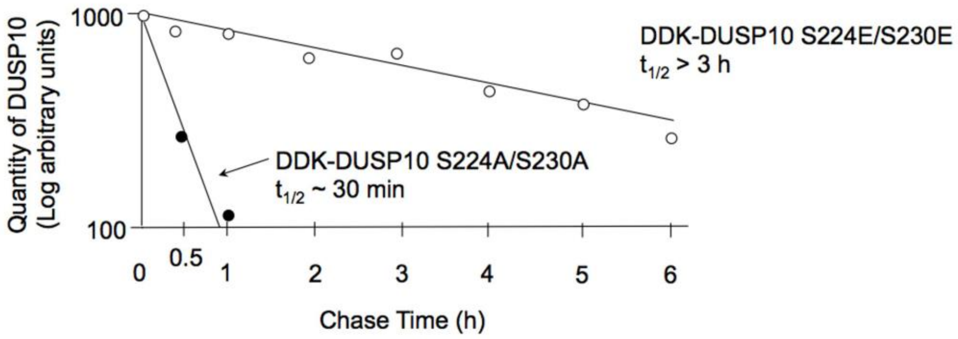

Supplementary Figure S2. Basal half-lives of S224/S230 DUSP10 mutants overexpressed in U87 cells. Closed circles correspond to values obtained for the nonphosphorylatable DUSP10 and the open circles correspond to values obtained for the phosphomimetic DUSP10 mutants as indicated.
